# Annotations

**Published:** 1898-05-14

**Authors:** 


### Annotations.

### The Midwives Bill.

The arrangement by which the Irish Local Government Bill is being taken *de die in diem* (except on Fridays) has deprived the promoters of the Midwives Registration Bill of the opportunity which would otherwise have been afforded them of making progress with that measure on Wednesday, and the probabilities are that no further chance will occur of obtaining a discussion of the subject by the House of Commons during this session. It must not, however, be imagined that no progress has been made in the settlement of this most urgent matter of providing better attendance upon lying-in women among the poor. On the contrary, the very fact that the Bill before Parliament appeared to have a real chance of becoming law has translated the question from academic regions into the arena of actual politics, and there can be no doubt that the real issue is much better and more widely understood than it was before. Little squabbles about titles, and puerile inventions of meaningless compound names cease to excite interest, and it is generally seen that the only question in which Parliament is at all likely to interest itself is the protection of poor women from the ghastly risks which they now run in their time of lying-in. We take it that the Bill is lost for this year, but if all the parties interested would but combine on the common platform that the real matter of importance is the protection of the lying-in woman who is too poor to afford medical attendance, and that the essential thing in any legislation is not the protection of a title, and not the making of high-class midwives, but the mere enforcement of a minimum of knowledge, without which the practice of midwifery shall be absolutely illegal—if, as we say, all would unite on such a platform, which surely all might agree to, we cannot but feel that, instead of a Bill for the regulation of midwives being looked at askance by all political parties, there would be an actual competition to get hold of it, for it would be a Bill which, without doing harm to any one, would tend to confer a definite and distinct benefit upon that great voting mass, "the working classes." Why cannot we come to some such common-sense basis of united action?

### School or Barrack?

There are 17,807 children under the care of the London Guardians. Of these a large majority are entirely brought up in what have been called "barrack" schools. The size of these varies greatly; some are arranged to hold only 100 children, and from this limit the capacity goes up to a school which is certified for 1,500. With the enormous number of children thrown upon their hands it is difficult to see what the Guardians can do but put them in school, give them good food and good education up to a certain age, and then send them out to the world, hoping that their children will not need to be maintained out of the rates. Perhaps this is the best that can be done; but if so, we fear that the best is bad. These barrack schools, however, do not seem to answer very well. "It seems strange," says a paper published by the "State Children's Aid Association," "that children who have the advantage of good food, suitable clothing, and palatial dwellings, should yet suffer from diseases consequent on malnutrition, but this fact has been stated and supported by medical men, in foreign countries as well as our own." It is more comprehensible that in these institutions the mental development of the pupils should be slow. Knowledge of the world is one of the things that comes only by observation, and education is apprehended most promptly by those who have to apply its teachings as they go along. In a large school the children are taken in the mass; it is impossible, however much the staff may wish it, to make discriminations between one and another; and the result is that all fall into a dead level of monotony. The spontaneous questions, the restlessness of childhood, must all be repressed, because they are contrary to discipline; and when you are dealing with masses, "discipline must be maintained." The only excitement which the children who are long in these schools get is derived from the experiences of some new-comer, whose knowledge of life is, nine times out of ten, exclusively of the seamy side. These bits of information, cherished all the more that they must be kept secret, form the stock-in-trade with which the child goes out into the world when his education comes to an end. Where will it lead him? It may be said that even larger numbers of children are handled in ordinary Board schools. True; but they are kept there, subject to discipline, only for a part of the day; the rest is spent in the freedom of home. And even a home that is not the best, in so far as it gives free play to individuality, is better than an institution. Discipline is, indeed, essential to the up-bringing of a child; but if he is to grow up into a free man, who acts on his own responsibility, freedom is not less essential. And freedom the best managed institution cannot, by reason of its institutional character, by any possibility give.

### The "Matching" of Milk.

Those who are concerned in the rearing of ailing and feeble infants by artificial feeding have lately laid much stress on the necessity of adjusting the composition of the milk employed to the digestive capacity of the individual child. No doubt, in the majority of cases, one or other of the two forms of humanised milk supplied by the Aylesbury Dairy Company answers the purpose pretty well; but with the view of enabling physicians to introduce greater individuality into their treatment, and thus to adapt the food to the infant with increased exactitude, the same company have arranged a department in which humanised milk is dispensed from physicians' prescriptions so as to contain any desired percentage of fat, milk sugar, and proteids. What may be the ultimate effect upon the race of rearing children whose digestive capacity is such that they cannot manage common food we will not suggest; but, so long as human nature remains as it is, each mother will fight for her own child and endeavour to rear it as well as she can, quite irrespective of sociological problems and abstruse questions in heredity, and there can be no doubt that the provision of special milks does give the modern mother great advantages in the rearing of children, who without such assistance might fail to get over that ticklish period in early infancy when the stomach is so apt to rebel against anything it does not like. In fact, to such a point is the preparation of special milks carried that mother's milk can now be "matched," as ladies would say of their ribbons, for the above-mentioned firm now undertake the analysis of any sample of human milk submitted by medical men, who are thus enabled to "match it" in their prescriptions. The possible utility of this proceeding when weaning has to take place early is obvious.

